# The Association between Serum Uric Acid Levels and 10-Year Cardiovascular Disease Risk in Non-Alcoholic Fatty Liver Disease Patients

**DOI:** 10.3390/ijerph19031042

**Published:** 2022-01-18

**Authors:** Kiduk Kim, Kyoonho Kang, Hyewon Sheol, Jihae Shin, Youngseo Sim, Taehoon Yang, Jeongwon Hwang, Ju-Mi Lee

**Affiliations:** 1 Eulji College of Medicine, Eulji University, Daejeon 34824, Korea; skdgh23@gmail.com (K.K.); rbsgh1229@naver.com (K.K.); khanhay11@gmail.com (H.S.); listenagain@naver.com (J.S.); deer227@naver.com (Y.S.); laverre@naver.com (T.Y.); awe7sov@gmail.com (J.H.); 2 Department of Preventive Medicine, Eulji College of Medicine, Eulji University, Daejeon 34824, Korea

**Keywords:** non-alcoholic fatty liver disease, uric acid, cardiovascular diseases, heart disease risk factors

## Abstract

Non-alcoholic fatty liver disease (NAFLD) and serum uric acid (SUA) levels are risk factors for developing cardiovascular disease (CVD). Additionally, previous studies have suggested that high SUA levels increase the risk of having NAFLD. However, no study has investigated the relationship between SUA and CVD risk in NAFLD. This study analyzed the relationship between SUA and CVD in NAFLD. Data for this study used the 2016–2018 Korean National Health and Nutrition Examination Survey, which represents the Korean population. A total of 11,160 NAFLD patients were included. Participants with hepatic steatosis index ≥ 30 were considered to have NAFLD. Ten-year CVD risk was estimated using an integer-based Framingham risk score. Estimated 10-year CVD risk ≥ 20% was considered high risk. Multiple logistic regression was conducted to calculate the odds ratios (ORs) associated with SUA level and CVD risk. High CVD risk OR increases by 1.31 (95% CI 1.26–1.37) times per 1 mg/dL of SUA. After adjustment, SUA still had an increased risk (OR 1.44; 95% CI 1.38–1.51) of CVD. Compared with the lowest SUA quartile group, the highest quartile group showed a significantly higher risk of having CVD before (OR 2.76; 95% CI 2.34–3.25) and after (OR 4.01; 95% CI 3.37–4.78) adjustment. SUA is independently associated with CVS risk in NAFLD.

## 1. Introduction

Non-alcoholic fatty liver disease (NAFLD) is defined as the accumulation of adipose cells in the liver, which promotes liver damage ranging from hepatic steatosis alone to cirrhosis, without alcohol consumption [1]. NAFLD is one of the most common chronic liver diseases globally, with an estimated prevalence of 10–20% in eastern countries and 20–30% in western countries [2]. Therefore, the substantial global economic burden could be caused by NAFLD, expected to be about 1 trillion dollars in the US and 334 billion euros in Europe [3]. Many factors are known to affect the development of NAFLD. Some genetic predispositions, including PNPLA3 and TM6SF2, are known to be associated with NAFLD [4,5]. Obesity [6], type 2 diabetes [7], and metabolic syndrome [8] are commonly known to be the risk factors for NAFLD. A variety of risk factors were proposed to develop NAFLD, and some recent studies suggested that serum uric acid is among them [9,10,11].

The disease burden caused by NAFLD is not only from the disease itself but also from indirect and elusive costs related to NAFLD. Chronic kidney disease, liver cancer, and cardiovascular disease could be associated with NAFLD. Among many diseases associated with NAFLD, cardiovascular diseases are responsible for most mortalities in NAFLD patients [12]. Cardiovascular diseases still remain one of the leading causes of mortality among Organization for Economic Co-operation and Development (OECD) member countries [13]. Many risk factors are known to be associated with the development of circulatory diseases. High blood pressure, dyslipidemia status, diabetic status, metabolic syndrome, smoking, and many other factors were suggested as the risk factors for cardiovascular disease [14]. Among many of these factors, serum uric acid was suggested as the important risk factor for vascular diseases. However, the question of whether the serum uric acid itself is the independent risk factor remains controversial [15].

Uric acid is the final metabolite of purine metabolism. Both prevalence and incidence of hyperuricemia are increasing in developed countries [16]. High serum uric acid levels are associated with various health conditions. Koudstaal et al. revealed that a high serum uric acid level increased the risk of myocardial infarction and stroke in the Rotterdam study [17]. Additionally, Obermayr et al. suggested elevated serum uric acid level as the risk factor for kidney disease [18]. There is substantial evidence suggesting that uric acid increases cardiovascular risk. However, it is not certain that uric acid per se is the risk for cardiovascular disease, or that is increases the risk of having mediating factors for developing cardiovascular disease. The Framingham heart study group contended that the cardiovascular outcome from serum uric acid attributed to other risk factors [19]. However, several attempts, including Santos et al. showed that uric acid is an independent risk factor for cardiovascular outcomes [20,21]. Additionally, Targher et al. suggested that uric acid can independently predict cardiovascular outcomes in type 2 diabetes [22].

Non-alcoholic fatty liver disease is one of the most common liver diseases globally, and the major burden of this disease is from the cardiovascular outcomes. Given that uric acid can be lowered using a uricosuric agent, and that it is a risk factor for both NAFLD and cardiovascular disease, the question of whether uric acid increases the risk of developing cardiovascular disease or not is important in NAFLD patients. However, no previous study has suggested the possible association between uric acid and cardiovascular disease in NAFLD patients. Therefore, we aimed to reveal the relationship between uric acid and cardiovascular disease in NAFLD patients in this study.

## 2. Methods

### 2.1. Study Participants

This study is based on the 2016–2018 Korea National Health and Nutrition Examination Survey (KNHANES) data. KNHANES is a cross-sectional study conducted by Korean Centers for Disease Control and Prevention (KCDC). The survey population is comprised of representatives of non-institutionalized citizens of Korea. A multi-stage clustered probability design was adopted to sample the representative population [23]. From 2016 to 2018, a total of 24,269 individuals were the study participants of the KNHANES. Among those individuals, 5290 participants with positive hepatitis B surface antigen, positive hepatitis C antibody, or those who have missing data for assessing infectious status were excluded. Additionally, 2207 participants were classified as high-risk drinkers (male: average alcohol consumption more than 7 units more than 2 times per week; female: average alcohol consumption more than 5 units more than 2 times per week) by KNHANES guidelines were excluded for excessive alcohol consumption [24]. After the exclusion, hepatic steatosis index (HSI) was used to evaluate the liver status. HSI was calculated according to the following equation: hepatic steatosis index (HSI) = 8 × AST⁄ALT ratio + BMI (+2 if DM; +2 if female).

**Figure 1:**
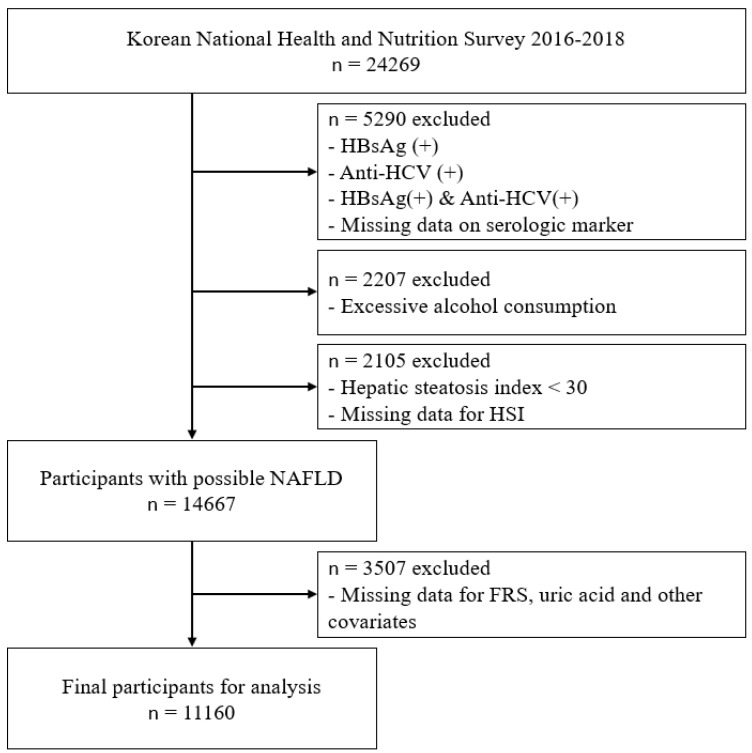
Study participants. Abbreviations: HBsAg—hepatitis B surface antigen; Anti-HCV—hepatitis C antibody; HSI—hepatic steatosis index; NAFLD—non-alcoholic fatty liver disease; FRS—Framingham risk score.

Since an HSI level under 30 can rule out the possibility of NAFLD [25], 14,667 individuals whose HSI level was 30 or over were considered to have NAFLD. Final 11,160 participants were eligible for the analyses after the additional exclusion for those missing data for assessing Framingham risk score, serum uric acid, and other covariates. (Figure 1)

### 2.2. Serum Uric Acid

Blood samples were obtained from a median cubital vein or cephalic vein after an 8 h fast. Serum uric acid level (mg/dL) was measured via the uricase colorimetry method with a Hitachi Automatic Analyzer 7600-210 (Hitachi/JAPAN). Serum uric acid levels were divided into quartiles of the whole population. Serum uric acid quartiles were used as follows: <4.1 (Q1), 4.1–4.7 (Q2), 4.8–5.7 (Q3), ≥5.8 mg/dL (Q4).

### 2.3. Framingham Risk Score and the Cardiovascular Disease Risk Groups

Study participants’ 10-year cardiovascular disease (CVD) risk was estimated using Framingham risk score (FRS) according to D’agostino et al. [26]. Age, serum high-density lipoprotein cholesterol (HDL-C), total serum cholesterol (TC), and systolic blood pressure (SBP), according to the participants’ treatment status, smoking, and diabetic status, were used to calculate FRS. According to this integer-based score [26], study subjects whose 10-year cardiovascular disease risk was over 20% (FRS ≥ 15 points for male; FRS ≥ 18 points for female) were classified as a high CVD risk group, and the others were classified as a low CVD risk group. Serum HDL-C level was measured using the homogeneous enzymatic colorimetric method and serum TC level using the enzymatic method with a Hitachi Automatic Analyzer 7600-210 (Hitachi/JAPAN). Participants were asked “Are you currently treated for hypertension?” to assess the treatment status of hypertension. SBP was measured three times, and the mean SBP of the second and third values was used. Those who smoked 100 cigarettes or more in a lifetime and were currently smoking were current smokers, and those who smoked 100 cigarettes or more in a lifetime and were currently not smoking were past smokers, and the others were grouped into never smokers. Current smokers were classified as smokers in calculating FRS, while past smokers and never smokers were non-smoker in calculating FRS. Fasting plasma glucose (FPG) level was assessed to evaluate the diabetic status via hexokinase UV using a Hitachi Automatic Analyzer 7600-210 (Hitachi / JAPAN). Participants whose FPG level was 126 mg/dL or higher or who were diagnosed with diabetes mellitus by a doctor or were taking medications for diabetes were identified as diabetes mellitus.

### 2.4. Confounding Variables

During the study, sociodemographic and lifestyle-related health factors were obtained with standardized health interview questionnaires and standardized health examinations [23].

Age was categorized into 30–39, 40–49, 50–59, 60–69, and ≥70 years. Participants were categorized into four groups by educational status. Household income quartiles were also applied to measure the socioeconomic status of participants.

The physically active group included those who engaged in at least 150 min of moderate-intensity physical activity, 75 min of high-intensity physical activity, or an equivalent combination of physical activity per week [27].

Body mass index (BMI) was used to evaluate the obese status of participants. Participants with BMI < 25 kg/m^2^ was considered normal, while BMI ≥ 25 kg/ m^2^ was considered to be obese [28]. Hypertension was defined as (1) SBP ≥ 140 mmHg, (2) diastolic blood pressure (DBP) ≥ 90 mmHg, or (3) those who were taking antihypertensive medications. Subjects were asked “Were you diagnosed to be dyslipidemia by a doctor?” to assess the dyslipidemia status. Additionally, the chronic kidney disease status was appraised with the following question: “Were you diagnosed to have chronic kidney disease by a doctor?” Participants were also assessed for having metabolic syndrome (MetS) according to the International Diabetes Federation (IDF). According to IDF criteria, a person should have central obesity (defined as a waist circumference of ≥90 cm for males, ≥80 cm for females), and any 2 additional factors out of 4 factors were diagnosed Mets. These 4 factors were the following: increased triglyceride level (≥150 mg/dL), reduced HDL-C (<40 mg/dL for males, <50 mg/dL for females), raised blood pressure (SBP ≥ 130 mmHg or DBP ≥ 85 mmHg, or taking antihypertensive medication), and raised FPG (≥100 mg/dL or previously diagnosed DM) [29].

### 2.5. Statistical Analysis

All categorical variables are presented with frequencies and weighted percentages. Row percentage was suggested when comparing cardiovascular disease risk groups. Continuous variables are presented with means ± SD or medians and interquartile range (Q1–Q3). Rao-Scott χ2 test was applied to analyze the difference between categorical values since KNHANES was a complex sample design. *t*-test and analyses of variances (ANOVA) were conducted to estimate the differences in numerical values among interested groups. Multiple logistic regression was conducted to estimate the odds ratios (ORs) and 95% confidence interval (CI) and evaluate the association between serum uric acid level and having a high CVD risk. Serum uric acid level was evaluated as both continuous values and quartiles for logistic regression. The association between serum uric acid level and high CVD risk was assessed before and after adjusting confounding and mediating variables. Model 1 is the model which is adjusted for possible confounders, including educational status, household income, physical activity, obesity, and chronic kidney disease. Additional adjustment for possible mediators was also made. Variables used to calculate Framingham risk score, including age, gender, smoking status, hypertension, diabetic status, and dyslipidemia, were adjusted in model 2. SAS 9.4 (SAS Institute Inc., Cary, NC, USA) was used for all statistical analyses. A two-sided *p*-value of 0.05 was set to estimate the statistical significance.

### 2.6. Ethics

This study was carried out following the latest version (2013) of the Code of Ethics of the World Medical Association (Declaration of Helsinki) for research involving humans, and the Institutional Review Board of Eulji University approved the study protocol (EUIRB2020-061).

## 3. Results

### 3.1. Baseline Characteristics among Serum Uric Acid Quartiles

Baseline characteristics of participants according to serum uric acid quartiles are described in Table 1. The total number of participants was 11,160. The fourth quartiles are younger than the other quartiles. The fourth quartile contains individuals as follows: more men, highly educated people, physically active people, obese people, current smokers, higher household income, and hypertension patients. However, the fourth quartile is less likely to have been diagnosed with dyslipidemia and have diabetes mellitus. The proportion of the cardiovascular disease risk over 20% was increased with serum uric acid quartiles (9.9%—Quartile 1; 12.8%—Quartile 2; 17.4%—Quartile 3; 23.3%—Quartile 4).

### 3.2. Baseline Characteristics of the Participants according to the 10-Year Cardiovascular Disease Risk

Table 2 shows the characteristics of the participants according to 10-year cardiovascular disease risk. The low CVD risk group was defined as an estimated cardiovascular disease risk of less than 20% (<20%). The high CVD risk group was defined as an estimated cardiovascular disease risk of equal to or more than 20% (≥20%) [30]. The high CVD risk group contains individuals of older age, more men, less educated, lower household income, less physically active, obese, and smokers. Additionally, the high CVD risk group shows more people with diagnosed chronic kidney disease, diabetes mellitus. The median serum uric acid level was higher in the high CVD risk group (5.3 mg/dL) than the low CVD risk group (4.7 mg/dL). Additionally, the high CVD risk group has more people in the highest quartile than the low CVD risk group.

### 3.3. The Association between Serum Uric Acid Level and Having a High CVD Risk

The association between serum uric acid level (per 1 mg/dL) and having a high CVD risk accessed by logistic regression are shown in Table 3. Model 1 showed the association after adjusting educational status, household income, physical activity, obesity, and chronic kidney disease. Although the Framingham risk score estimation formula already considered age, gender, smoking status, dyslipidemic status, and hypertensive and diabetic status, we ran additional adjustments. Additional adjustment for the factors related to Framingham risk score (possible mediating factors: age, gender, smoking status, dyslipidemic status, and hypertensive and diabetic status) was conducted, and the results are shown on model 2. In unadjusted analysis, serum uric acid level (per 1 mg/dL) was significantly associated with increased odds of having a high CVD risk (OR 1.31, 95% CI 1.26–1.37, *p* < 0.001). In model 1, the odds of having a high CVD risk were 1.44 (95% CI 1.38–1.51, *p* < 0.001) after adjustment of confounders. After the additional FRS component adjustment, model 2 showed odds of high CVD risk of 1.10 (95% CI, 1.02–1.19; *p* = 0.018).

Table 4 showed the association between serum uric acid quartile groups and having a high CVD risk. Compared with the 1st serum uric acid quartile group (Q1), the ORs of having a high CVD risk for the 2nd, 3rd, and 4th quartile groups (Q2, Q3, Q4) were statistically significant. After adjusted the confounders in model 1, OR was 1.43 (95% CI 1.18–1.72, *p* < 0.001) at Q2, 2.26 (95% CI 1.90–2.69, *p* < 0.001) at Q3, and 4.01 (95% CI 3.37–4.78, *p* < 0.001) at Q4 compared with Q1. The strength of the association (ORs) between serum uric acid quartile and having a high CVD risk were increased as the uric acid quartile increase in model 1 and the unadjusted model. Associations were slightly weakened after the additional adjustment for the factors related to the Framingham risk score.

The ORs of having a high CVD risk compared with the highest serum uric acid quartile (4Q) are shown in Supplementary Table S1. The association was statistically significant at Q1, Q2, and Q3 compared with Q4 in model 1 and the unadjusted model. The strength of the association (ORs) between serum uric acid quartile and having a high CVD risk were decreased as the uric acid quartile decreased in model 1 and the unadjusted model. After the additional adjustment for the factors related to the Framingham risk score, all significance was lost except Q1. However, Q2 and Q3 showed lower OR for having a high CVD risk than Q4.

## 4. Discussion

We examined the association between high CVD risk and serum uric acid levels in non-alcoholic fatty liver disease patients in this study, using population-representative data of Korea. The odds of having CVD risk ≥ 20% increased by 1.31 times for each 1 mg/dL of serum uric acid level. This result maintained the adjustment of the possible confounding factors. After adjusting for confounding factors, the odds increased 1.44 times for each 1 mg/dL of serum uric acid level. Although the Framingham risk score estimation formula already considered age, gender, smoking status, dyslipidemia, hypertension, and diabetic status, we ran additional adjustments. The odds of CVD risk ≥ 20% increased 1.10 times for each 1 mg/dL of serum uric acid level after excluding mediating effect. Additionally, compared with the first serum uric quartile group, the risk of having high CVD risk increased 1.43 times with the 2nd quartile group, 2.26 times with the 3rd, and 4.01 times with the 4th quartile group, after the adjustment of possible confounding factors.

This was the first study to present that serum uric acid is an independent risk factor for cardiovascular disease in non-alcoholic fatty liver patients. In the general population, the relationship between uric acid and cardiovascular disease still remains undetermined [15]. In 1999, The Framingham heart study group contended that serum uric acid is not the independent risk factor for the development of cardiovascular disease but rather associated with other risk factors [19]. However, several recent studies suggested that serum uric acid might be the risk factor for cardiovascular disease risk. Gang et al. proposed that baseline serum uric acid level could be an independent predictor of cardiovascular disease mortality, especially in men [31]. A prospective study with a 21-year follow-up suggested that serum uric acid is an independent predictor for major cardiovascular death in older women [32]. There was also an attempt to prove the causal relationship between serum uric acid and cardiovascular events using a mendelian randomization study design [33]. Some subgroup studies prove that serum uric acid is an independent risk factor for cardiovascular disease. Evidence suggests serum uric acid as a marker for cardiovascular disease risk in a healthy population [34]. The subgroup analysis for rheumatoid arthritis patients also suggested that serum uric acid might be an independent risk factor for cardiovascular disease in this group [35].

There might be some plausible explanations that can convince this association in NAFLD patients. Uric acid is the final product of purine metabolism. Therefore, uric acid might be a marker for purine metabolic activity. CARES trial revealed that allopurinol had a more protective effect over febuxostat [36]. Given the fact that allopurinol is a purine analog, febuxostat is a non-purine xanthine oxidase inhibitor, and allopurinol has a broader effect among purine metabolism [37,38], active purine metabolism per se or other by-products of purine metabolic activity might have affected the cardiovascular risk in NAFLD patients. Additionally, it is known that xanthine oxidoreductase shows the most vigorous activity in the human liver [39]. Therefore, oxidative stress generated by uric acid [40] might affect NAFLD’s progression [41]. Given that NAFLD can exert a causal effect on cardiovascular disease via insulin resistance or systemic inflammation [42], and that NAFLD and cardiovascular disease share many parts of underlying mediators and risk factors [43,44,45], the progression of NAFLD might have increased the cardiovascular disease risk in high serum uric acid group.

Liver biopsy is the gold standard for diagnosing NAFLD [46], but it is an invasive procedure, and can have the complication of harming patients, which is dependent on the operator’s experience [47]. Therefore, non-invasive ultrasound is widely used and recommended in diagnosing NAFLD [48,49]. However, the diagnosis with ultrasound also largely depends on the practitioner’s experience, and not all clinics are equipped with ultrasound. In addition, ultrasound can underestimate the prevalence of NAFLD when the patients’ fat occupies the liver under 20% [50]. We adopted the hepatic steatosis index (HSI) to diagnose NAFLD in this study. HSI uses simple parameters, such as gender, diabetic status, BMI, and liver enzymes, to screen NAFLD with the area under the receiver-operated characteristics curve of 0.812 [25]. Therefore, it can be easily and convincingly used in primary clinics with general practitioners.

We adopted the Framingham risk score for predicting 10-year cardiovascular outcomes for the participants. Among many other measures which can reflect cardiovascular risks, Framingham risk score, an integer-based risk assessment tool, can be easily used in primary settings [26]. In 2013, the American Heart Association invented the “2013 ACC/AHA Guideline on the Assessment of Cardiovascular Risk” to predict cardiovascular outcomes more accurately [51]. However, this current guideline did not provide the stratified score for Asian populations. Some previous studies suggested that pooled cohort risk scores from the 2013 ACC/AHA guideline might overestimate the cardiovascular risk in Asian populations [52,53]. Framingham general cardiovascular risk score accurately estimates the cardiovascular outcome among Asian populations [54,55]. We adopted the Framingham risk score for this current study for those reasons above. Additionally, several previous studies applied this scoring system to predict the 10-year cardiovascular disease risk. Using the Framingham risk score, Al-Khalidi et al. revealed the relationship between vitamin D and cardiometabolic disease risk [56]. Færch et al. described the possible association between cardiovascular risk and hyperglycemia concerning insulin resistance using the Framingham risk score [57]. Additionally, some approaches were associated with sarcopenia with cardiovascular risk, adopting this score [58,59,60]. Our study is in line with those previous studies, seeking out cardiovascular disease risk factors.

This present study has some strengths. First of all, various parameters and scores we used in this study design, such as the hepatic steatosis index, serum uric acid, and the Framingham risk score, which can be widely adopted in primary care without much difficulty. Second, this is the first study describing the association between serum uric acid and cardiovascular risk in relation to the progression of non-alcoholic fatty liver disease using nationally representative data. Third, the study results were consistent before and after adjusting the possible confounders and mediating factors, including metabolic syndrome and each factor of the Framingham risk score. Therefore, we could describe the effect of serum uric acid on cardiovascular risks by means of the integrated effect of mediators and independent effects.

Meanwhile, some limitations still exist in this study. It is based on the observational, cross-sectional survey; therefore, we cannot claim direct causality for these results. We used the Framingham risk score for the evaluation of cardiovascular risk. This index might be imprecise compared with other cardiac evaluation measures, such as coronary calcium score or cardiac sonography. Likewise, hepatic steatosis index cannot precisely diagnose non-alcoholic fatty liver disease compared with ultrasound or liver biopsy. Therefore, this study might overestimate the non-alcoholic fatty liver disease populations. Moreover, the study population in this report can represent the Korean population, but the results might not be generalizable to other ethnicities. Further prospective studies covering other ethnicities are needed to confirm these study results.

## 5. Conclusions

This study suggests that serum uric acid level correlated with statistical significance with 10-year cardiovascular risk in non-alcoholic fatty liver disease patients. Moreover, high serum uric acid quartiles were also associated with a high 10-year cardiovascular disease risk. The association was maintained after the adjustment of possible confounders and mediators. Further multi-ethnic prospective studies are needed to validate these study results.

## Figures and Tables

**Table 1:** Baseline characteristics of the participants according to serum uric acid quartiles.

Variables	Quartile 1 (*n* = 3089)	Quartile 2 (*n* = 2537)	Quartile 3 (*n* = 2906)	Quartile 4 (*n* = 2628)	*p*-Value
Age (years)	52.8 ± 0.2	53.1 ± 0.3	53.8 ± 0.3	51.9 ± 0.3	<0.001
30–39	496 (18.3)	420 (19.7)	440 (18.8)	511 (25.4)	<0.001
40–49	713 (27.4)	518 (23.3)	526 (21.8)	492 (23.0)	
50–59	671 (16.4)	569 (25.1)	649 (25.9)	481 (21.3)	
60–69	638 (16.4)	538 (17.8)	640 (17.6)	512 (14.9)	
≥70	571 (14.2)	492 (14.1)	651 (15.9)	632 (15.4)	
SEX Female	2745 (88.3)	2067 (78.6)	1731 (54.8)	637 (19.3)	<0.001
Education ≤Elementary school	767 (20.6)	569 (17.7)	627 (16.3)	480 (12.4)	<0.001
Middle school	382 (10.6)	343 (13.2)	387 (11.4)	283 (8.8)	
High school	872 (31.0)	699 (30.2)	829 (29.9)	706 (26.8)	
≥College	1068 (37.8)	926 (38.9)	1063 (42.5)	1159 (52.0)	
Household income (quartile)					<0.001
1/4	654 (17.9)	502 (16.6)	598 (16.6)	538 (14.9)	
2/4	750 (23.5)	652 (26.0)	708 (23.6)	641 (22.6)	
3/4	840 (29.3)	711 (30.5)	768 (28.3)	688 (29.0)	
4/4	845 (29.2)	672 (26.9)	832 (31.5)	761 (33.4)	
Physical activity Yes	353 (12.2)	334 (12.9)	408 (15.9)	400 (17.2)	<0.001
Obesity (BMI, kg/m^2^)	23.1 ± 0.1	23.8 ± 0.1	24.5 ± 0.1	25.7 ± 0.1	<0.001
Normal (<25)	2329 (75.7)	1727 (68.3)	1692 (59.5)	1194 (43.1)	<0.001
Obese (≥25)	760 (24.3)	810 (31.7)	1214 (40.5)	1434 (56.9)	
Dyslipidaemia Yes	659 (18.9)	568 (19.8)	670 (21.5)	568 (19.2)	0.105
Chronic kidney disease No	3086 (99.9)	2533 (99.9)	2898 (99.8)	2612 (99.6)	0.026
Smoking status					<0.001
Never smoker	2671 (86.0)	2014 (76.7)	1875 (62.0)	1128 (41.7)	
Past smoker	256 (8.0)	288 (11.5)	655 (23.1)	914 (32.9)	
Current smoker	162 (6.0)	235 (11.8)	376 (14.9)	586 (25.4)	
Hypertension					0.001
Yes	925 (26.1)	798 (27.7)	1083 (33.1)	1189 (40.4)	
Diabetes mellitus					0.019
Yes	430 (12.5)	331 (11.9)	471 (15.0)	422 (13.0)	
Cardiovascular disease risk					<0.001
≥20%	368 (9.9)	388 (12.8)	647 (17.4)	813 (23.3)	
<20%	2721 (90.1)	2149 (87.2)	2259 (82.6)	1815 (76.7)	

BMI—body mass index.

**Table 2:** Baseline characteristics of the participants according to 10-year cardiovascular disease risk.

Variables	Low CVD Risk Group (<20%) (*n* = 8944)	High CVD Risk Group (≥20%) (*n* = 2216)	*p*-Value
Age (years)	49.9 ± 0.1	68.7 ± 0.2	<0.001
30–39	1867 (100)	0 (0)	<0.001
40–49	2222 (98.4)	27 (1.6)	
50–59	2136 (88.9)	234 (11.1)	
60–69	1729 (73.0)	599 (27.0)	
≥70	930 (44.1)	1356 (55.9)	
Sex Female	6489 (92.4)	691 (7.6)	<0.001
Education ≤Elementary school	1502 (64.0)	941 (36.0)	<0.001
Middle school	994 (74.0)	401 (26.0)	
High school	2580 (86.2)	526 (13.8)	
≥College	3868 (93.1)	348 (6.9)	
Household income (quartile)			<0.001
1/4	1342 (62.9)	950 (37.1)	
2/4	2183 (83.3)	568 (16.7)	
3/4	2605 (89.3)	402 (10.7)	
4/4	2814 (91.3)	296 (8.7)	
Physical activity Yes	1314 (90.3)	181 (9.7)	<0.001
Obesity (BMI, kg/m^2^)	24.2 ± 0.0	24.9 ± 0.1	<0.001
Normal (<25)	5749 (86.5)	1193 (13.5)	<0.001
Obese (≥25)	3195 (80.2)	1023 (19.8)	
Dyslipidemia Yes	1737 (73.5)	728 (26.5)	<0.001
Chronic kidney disease No	8925 (84.2)	2204 (15.8)	<0.001
Smoking status			<0.001
Never smoker	6764 (90.4)	924 (9.6)	
Past smoker	1341 (73.0)	772 (27.0)	
Current smoker	839 (69.8)	520 (30.2)	
Hypertension Yes	2307 (62.0)	1688 (38.0)	<0.001
Diabetes mellitus Yes	674 (45.2)	980 (54.8)	<0.001
Serum uric acid (quartile)	4.7 (3.9–5.7)	5.3 (4.4–6.3)	<0.001
1/4	2721 (90.1)	368 (9.9)	<0.001
2/4	2149 (87.2)	388 (12.8)	
3/4	2259 (82.6)	647 (17.4)	
4/4	1815 (76.7)	813 (23.3)	

CVD—cardiovascular disease; BMI—body mass index.

**Table 3:** The association between serum uric acid level and having a high CVD risk.

	Crude Model		Model 1		Model 2	
	OR (95% CI)	*p*-Value	OR (95% CI)	*p*-Value	OR (95% CI)	*p*-Value
Serum uric acid (per 1 mg/dL)	1.31 (1.26–1.37)	<0.001	1.44 (1.38–1.51)	<0.001	1.10 (1.02–1.19)	0.018

Model 1: adjusted for educational status, household income, physical activity, obesity, and chronic kidney disease. Model 2: adjusted for age, gender, smoking status, hypertension, diabetic status, and dyslipidemia at model 1.

**Table 4:** The association between serum uric acid quartile and having a high CVD risk.

Serum Uric Acid (Quartile, mg/dL)	Crude Model		Model 1		Model 2	
	OR (95% CI)	*p*-Value	OR (95% CI)	*p*-Value	OR (95% CI)	*p*-Value
Q1 (<4.1)	Ref.		Ref.		Ref.	
Q2 (4.1–4.7)	1.33 (1.11–1.60)	0.002	1.43 (1.18–1.72)	<0.001	1.38 (1.03–1.86)	0.033
Q3 (4.8–5.7)	1.91 (1.62–2.25)	<0.001	2.26 (1.90–2.69)	<0.001	1.19 (0.90–1.59)	0.227
Q4 (≥5.8)	2.76 (2.34–3.25)	<0.001	4.01 (3.37–4.78)	<0.001	1.44 (1.07–1.94)	0.017

Model 1: adjusted for educational status, household income, physical activity, obesity, and chronic kidney disease. Model 2: adjusted for age, gender, smoking status, hypertension, diabetic status, and dyslipidemia at model 1.

## Data Availability

Korean National Health and Nutrition Examination Survey is publicly available dataset. This data can be found here: https://knhanes.kdca.go.kr/knhanes/sub03/sub03_02_05.do/accession (accessed on 13 December 2021) number not required.

## Supplementary Materials

The following supporting information can be downloaded at: https://www.mdpi.com/article/10.3390/ijerph19031042/s1, Table S1: The association between serum uric acid quartile and having a high CVD risk.
