# Supplementary material for: The Association between Serum Uric Acid Levels and 10-Year Cardiovascular Disease Risk in Non-Alcoholic Fatty Liver Disease Patients
Source: Int J Environ Res Public Health. 2022 Jan 18;19(3):1042. doi: 10.3390/ijerph19031042 (PMC8834479; doi:10.3390/ijerph19031042)
Supplement: Supplementary file 1 [file ijerph-19-01042-s001.zip › ijerph-1531492-supplementary.pdf]

Supplementary Table S1. The association between serum uric acid quartile and having a high CVD risk

Serum uric acid (quartile, mg/dL)	Crude model		Model 1		Model 2	
	**OR (95% CI)**	***p*-value**	**OR (95% CI)**	***p*-value**	**OR (95% CI)**	***p*-value**
Q1 (<4.1)	0.36 (0.31–0.43)	<0.001	0.25 (0.21–0.30)	<0.001	0.70 (0.52–0.94)	0.017
Q2 (4.1–4.7)	0.48 (0.41–0.57)	<0.001	0.36 (0.30–0.42)	<0.001	0.96 (0.73–1.26)	0.761
Q3 (4.8–5.7)	0.69 (0.60–0.80)	<0.001	0.56 (0.48–0.66)	<0.001	0.83 (0.66–1.05)	0.115
Q4 (≥5.8)	Ref		Ref		Ref	

Model 1: adjusted for educational status, household income, physical activity, obesity, and chronic kidney disease. Model 2: adjusted for age, gender, smoking status, hypertension, diabetic status, and dyslipidaemia at model 1.
